# On Paracentesis Thoracic in Pleuritic Effusion

**Published:** 1854-10

**Authors:** 


*On Paracentesis Thoracic in Pleuritic Effusion.* By (1) Dr. Bowditch, Physician to the Massachusetts General Hospital; and (2) Dr. Tracy, of Hollis (U. S.)

(1) *American Medical Monthly*, Jan. and Feb., 1854; *New York Journal of Medicine*, Nov., 1853.

1. Dr. Bowditch's communication, which is a continuation of a former paper on the subject (vide *Abstract*, vol. xvi.) is an analysis of 25 cases, in which he either operated himself or saw others operate.

In his former paper eight of these cases are narrated, and certain practical results arrived at, which are expressed in the following words:—

"I shall puncture the chest; *first*—whenever, either in an acute or chronic case, I find a pleural cavity *distended* or *filled* with fluid; *second*—whenever, in any *acute* case, remedies seem to have but little effect toward causing an absorption of the fluid, and after a fair trial has been made of them for two, three, or four weeks; *third*—I shall puncture in cases of larger effusion, complicated with organic disease, in the hope of relieving urgent dyspnœa or to lengthen life."

Now Dr. Bowditch says—

"I would not wait so long as 'three or four weeks' in acute attacks, provided I found that the effusion continued steadily to augment in spite of remedies. Moreover, if called in an acute case that has lasted a month, and in which there is an amount of fluid effused, sufficient to materially compress the lung, I shall advise a puncture, as the *first* step to be taken, previously to the use of the remedies commonly employed in pleuritic affections."

The 25 cases are numbered chronologically, and classed in four main divisions, according to the effect of the operation.

*First class*, or those cases in which the operation has been the chief or sole cause of the cure of the pleuritic effusion. Cases 1, 7, 8, 11, 12, 14, 15, 18, 20, 21, (total, 10,) are of this class.

*Second class*, or those cases in which the puncture has given more or less, and at times very great, temporary relief, so that some of the patients have asked for the operation a second, third, or fourth time, for the sole object of getting relief. Cases 2, 3, 4, 9, 10, 13, 16, 17, 19, (total, 9,) are of this class.

*Third class*, or those in which no relief was obtained, because no fluid could be removed. Cases, 5, 6, and 25, (total, 3,) are of this class.

*Fourth class*, or those still under treatment, which are progressing favorably, with more or less rapidity. Cases 22, 23, 24, (total, 3,) are of this class.

The cases subjoined belong respectively to the first and second classes. They are taken indiscriminately and without any selection.

CASE 15.—June 10th, 1853. Mrs. B—, I saw at E. Boston, with Dr. ——; æt. 45. She was the mother of a large family, which she had usually superintended until her illness; but she had been considered tuberculous, and for months had used cod-liver oil, under which, previously to her actual attack, she had been tolerably well. For three months before I saw her, she had felt not quite so well. Six weeks previously she had had an acute pain in the right side; but it did not prevent her from going about the house. Three weeks before the operation, she went to church all day. While dressing for this purpose, she was surprised to find that her gown was too tight, and she had some dyspnœa. She, however, continued at work for a few days, when, owing to an increase of the symptoms, she was compelled to desist; she lost her appetite; the cough became dry and hard; the dyspnœa was extreme, so that at length she could not get up into her chamber, and fits of suffocation occurred, threatening death.

I found her with an anxious, very livid countenance, in bed, half erect, pulse 115. Respiration much labored. On percussion, the right side was flat everywhere, except at the apex behind. Respiration scarcely heard, even under the clavicle; bronchial for a small space along the vertebræ from top to bottom, absent elsewhere. Puerile through the whole of the left.

I punctured between the eighth and ninth ribs behind, and drew eighty-three and three-fourths ounces of yellow serum. The patient experienced the greatest possible relief, and suffered scarcely at all, except at the last of the operation she had some stricture across the chest, and the cough was a little troublesome. The sounds on percussion *instantly* became more clear to the point of puncture. The bronchial respiration was replaced by the vesicular. Crackling was heard throughout the right breast, evidently from the expanding lung. The pulse fell to 108, and she was able to lie on the left side, in a position which nearly suffocated her only twenty minutes before the operation. She was allowed broth and wine. During the next twenty-four hours, she coughed much, and raised nearly a quart of frothy white fluid.

I saw her, P. M., June 11th, and found her quiet, with much more easy breathing; she was much less livid; she relished her broth and wine.

From this time, she steadily progressed, the lung expanded rapidly, as marked by râles that were heard everywhere in it. The little fluid that remained in the pleural cavity was soon absorbed; the urine was much increased. The œdema of the legs, that existed before the operation, was wholly gone by the 20th (ten days after operation;) the lividity of the skin had subsided. The appetite and digestive functions improved, and were excellent at the above date; no dyspnœa; pulse 85, quiet; little cough; only felt weak. On percussion (20th,) tenth day from puncture, there was only a difference of pitch between the two sides—no real dullness. Vesicular murmur was heard in every part, only a little less at the right than at the left, with a dry crackle at the top on coughing.

Sept. 23d. I found she had been going on well, though she was still feeble; scarcely any cough; digestion excellent; slight feeling as of pain or obstruction in the right side on full breath; was able to superintend her domestic affairs; she visited me at my office. On percussion, less sound at the *left* than the *right* top, and the voice was more resonant there; and I thought I heard, at times on coughing, a slight crackle there. Murmur obscure at the right apex. *Equal and clear in both lower lobes.* In other words, the signs were those of the previous chronic disease, the acute pleurisy having left little or no trace of its existence. Ordered to resume cod-liver-oil.

CASE 16.—Mr. H.—entered Massachusetts General Hospital, May 28, 1853. Sick three months only; he first noticed a cough, which came on after an exposure to wet and cold—no hæmoptysis. He was very ill at his entrance into the hospital, and continued to grow worse, with signs of disease in both cavities of the thoràx. Dullness on percussion was observed in the lower part of both backs; the respiration was rude and bronchial at the left; mucous and sonorous râles everywhere. He was supposed to have pleurisy of both sides, and disease of the lungs, probably tuberculous. On 11th of July, the report by Dr. Storer was as follows: "Has been gradually failing during the last week; greater dyspnœa; at each visit bathed in cold sweat; countenance haggard, although he constantly reports himself as comfortable; pulse usually was 120." On this day I operated, at the request of Dr. Storer, between the ninth and tenth ribs, and drew off twenty-three ounces of highly colored serum. On the subsequent day the record was—"Comfortable day and night; respiration less labored; pulse 110; countenance more tranquil." He continued improving until the 17th, when the dyspnœa was augmented. He afterward grew worse; Aug. 5th, I punctured anew, and drew off thirteen ounces of colored serum. Little relief ensued, and he soon afterward left the hospital to die.

The conclusion from the clinical evidence is,—

"First, no one of the patients operated on experienced a single dangerous symptom, or any materially unpleasant symptom, except for a short period.

"Secondly, Out of twenty-five persons, only three failed of obtaining relief. Of these three, two had pulmonary (probably tubercular) disease; and from the other no fluid could be drawn, owing, perhaps, to an imperfection of the instrument which I used in my earlier operations.

"Thirdly, in more than half of the cases, the puncture was the first remedial agent that decidedly arrested the progress of the disease. This it did in two modes. 1st, by allowing the lung to expand immediately, and producing thereby a rapid cure. 2d, by so stimulating the functions of the body, made torpid by long disease, that they immediately sprang into healthful, vigorous action, while the lung expanded more slowly.

"That this stimulus which I have mentioned as occurring in the second class, actually takes place in many cases, I am sure. I have so repeatedly noticed it that I now confidently hope for its occurrence, when I do not find that a case, after a puncture, is likely to be of the first class. I do not mean to state that the stimulus shows itself immediately, or that it acts with rapidity in every case, but simply that from the moment of drawing off the fluid, I have been able to trace a series of favorable influences tending toward health.

"Fourthly, In about *seven-eighths* of the cases, the operation has given *great relief* to prominent and distressing symptoms, insomuch that the patients have asked for a second, third, or fourth puncture, as a means of relief only."

The symptoms consequent upon the puncture were very similar to those reported in my former paper. The pain of the puncture was the chief trouble, and this, as it was momentary, was but little noticed by the majority. Stricture across the chest was occasionally noticed toward the end of the operation. The cough was augmented in many. This I regarded as a favorable sign, as it usually indicates that the compressed lung is beginning to expand. In one case this symptom was excessive, it having lasted twenty-four hours almost without intermission. In this case the lung arose instantly from its compression. One had vomiting of her dinner, the operation having been done in the afternoon. In all, where fluid was obtained, the oppression was somewhat relieved; in one, impending suffocation was prevented. Most of the patients were exhilarated by the success of the operation, as in our previous set of cases. In one, there was a slight oozing of blood from the point of puncture, which, however, was easily checked.

"The *pulse* remained tranquil, as much as it was before the operation.

"The *digestive* functions were improved. In all, where much fluid was obtained, the appetite was improved with singular rapidity. One person asked for food before we left the house.

"The *urine* was augmented frequently by the operation, a fact which I noticed often when analyzing the first set of cases.

"In *none* was the fever augmented, or a febrile paroxysm excited.

"The physical signs altered slowly in some cases, in others very rapidly. The patient in case 15, having been ill a few weeks, presented the phenomenon of the lung completely expanded and filled with râles the next day after the removal of five pints of fluid. Generally, however, a more slow process was carried on, the lung expanding in the first few hours only along the vertebræ and at the apex, and thence more or less gradually rising to meet the parietes of the chest; the parts under the axilla being, of course, the last to fill out. In some instances, that state of the lung described by Dr. Gairdner, remained for months, the patients being nearly free from all rational symptoms of disease, save, perhaps, a tendency to dyspnœa."

The character of the fluid drawn from the chest varied. By a reference to the tabular statement, it will be seen that from forty-seven punctures, the following results were obtained:

TABLE II.

| | |
|---|---|
| Nothing, . . . . . . . . . | 5 times. |
| Serum, a few drops only (2 cases,) . . | 4 " |
| Serum in large quantities, . . . . . | 16 " |
| Pus, or purulent, . . . . . . . | 17 " |
| Bloody, . . . . . . . . . | 5 " |

The *quantity* of the fluid varied considerably; three ounces being the smallest, one hundred and seventy ounces being the largest. In this latter case it was pure pus.

The influence of the character of the fluid, the length of the disease previous to the operation, and the existence or non-existence of previous disease, may be learned by the following series of tables:

TABLE III.

| Character of the Fluid in the Chest. | Serum. | Pus. | Bloody. | Total. |
|---|---|---|---|---|
| Recovery from pleuritic effusion, . . . | 7 cases, | 5 cases, | 1 case, | 13 cases. |
| Death afterward, consequent upon the effusion and previous disease, . . . | 3 " | 4 " | . . | 7 " |
| Friction sound heard, but death a few weeks after from disease of brain, . | 1 " | . . | . . | 1 " |
| Under treatment, doing well, . . . | 1 " | . . | . . | 1 " |
| Under treatment with prospect of months of illness, . . . . . . . , . . . | | 2 " | . . | 2 " |
| | | | | 24 cases. |

"It seems, therefore, that the presence of the serum is more favorable for the prognosis than is the existence of pus. This only confirms our preconceived notions, but it is rather different from the opinion I advanced in my previous paper, the facts contained therein not allowing me to hold the opinion I now advance."

The next important element in the prognosis is the length of time the disease has lasted previous to the operation. The following table will show this:—

TABLE IV.

| | | Serum. | Pus. | Bloody. |
|---|---|---|---|---|
| Average time before puncturing in cases of | recovery, . | 2½ months, | 2 months, | |
| | death, . . | 3 " | 4½ " | 3 months. |

Whence it appears that whether pus or serum exists, an early operation is more favorable than a later one.

The influence of the existence or non-existence of previous disease may be illustrated by the following:—

TABLE V.

| | Of those who had no disease immediately preceding the effusion. | Of those who had cough, and were probably phthisical. |
|---|---|---|
| Recovered from the effusion, . . | 10 . . . . | 4 |
| Died with effusion remaining. . . | 0 . . . . . | 6 |

"From this table we infer, what, in fact, we knew before, that pleuritic effusions, uncombined with serious pulmonary disease, do not usually destroy life. I cannot but think, however, that in case No. 2 the operation may be said to have saved life, for a time, at least. In case 15 I have no doubt suffocation would have taken place, had not the operation been performed.

"Another interesting inference is suggested by this table, viz: we observe that of 10 who had organic diseases, 4 were cured of the pleuritic effusion, 6 died. Now the puncture was the *sole* cause of the cure of these four, for the lung expanded in all of them within twenty-four hours or a few days after the operation was done. No other cause operated, and therefore to the thoracentesis we must attribute the cure. Is there any physician that can say as much of any other method of cure under similar circumstances? Is there any remedy which will cause an absorption of five pints of fluid in twelve hours, and allow a lung that has been compressed for months to be thoroughly filled with air in twenty-four hours?

"In confirmation of these remarks, and to give the reader a more definite idea of the *amount* of influence the puncture had toward the *cure* or *relief* of the effusions, I submit the following data taken from my own cases, compared with similar data obtained by the courtesy of Mr. Scarem, at present house-pupil of the Massachusetts General Hospital, from the records of that institution. In preparing my own, I have taken, *first*, all those cases in which the lung, after having been for weeks, or perhaps for months compressed, has suddenly expanded, within twelve or twenty-four hours after the puncture; *second*, those in which the stimulus above spoken of was given to the various functions of the body, so that all the rational signs grew decidedly better from the moment the fluid was evacuated, while the long-compressed lung dilated but slowly.

"In the first, the lung expanded immediately, or within twelve hours after the puncture. In the second, the lung, on an average, in $32\frac{1}{2}$ days, or $4\frac{4}{7}$ weeks after the puncture.

"I think no one can doubt that paracentesis *cured* the disease in the first class of cases. In proof that it aided very materially toward the same results in the second class, I present the subjoined table of comparison between my cases and and those treated at the Hospital.*

---

* This table is founded on data drawn from fifty-four cases of pleuritic effusion found recorded in the books of the Hospital, between Jan. 4, 1847, and Sept. 9, 1853. In it I have made use of those cases only, in which the disease could be traced by the rational and physical signs to its termination in the Hospital; or, if the patient left the Hospital before recovery, but after a *long* residence at the institution, I have added the sign + to the number of months the case was under the care of the institution. From my own cases, I have only taken those of a similar character, viz. Nos. 1, 7, 8, 11, 12, 15, 18, 20, 21.

TABLE VI.

| Length of time the disease lasted. | HOSPITAL CASES. Whole length of the disease. | After entering hospital. | MY CASES. Whole length of the disease. | After Thoracentesis. |
|---|---|---|---|---|
| Average duration in cases of complete filling of one pleural cavity . . . . | 12+weeks. | $6\frac{5}{8}$+weeks. | 13 weeks. | 3 weeks. |
| do. partial do. | 12 " | $6\frac{1}{2}$ " | $9\frac{1}{4}$ " | 4 " |

"Supposing all these data to be *absolutely* correct, I might draw from them the following propositions:

"1st. *One pleural cavity being full of fluid.*—*a.* Thoracentesis shortens the disease more than *one half.*

"2d. *One pleural cavity being partially filled.*—*b.* Thoracentesis shortens the disease more than *one third.*

"I do not, however, present them as absolutely correct, but merely as approximations to the truth. But I do not see that any one can deny, that puncturing the chest does very materially shorten, and consequently alleviate the sufferings of a patient affected with pleuritic effusion. As if in confirmation of this view, we see that although it appears, in my cases of complete filling of the pleural cavity, that the whole duration of the disease was perhaps as long as it was in the hospital cases, nevertheless there was this great difference of time after the two treatments were commenced, before the effusion was removed: viz., those treated by paracentesis getting well in half the time required by the hospital treatment. I do not believe, however, that thirteen weeks shows the duration of the disease as it will be when tapping is resorted to with as much freedom as we resort to calomel, blistering, &c. For this period of thirteen weeks is really owing to one case, which had lasted *seven months* before a puncture was made. Excluding this case from the calculation, we shall get $7\frac{3}{4}$ weeks as the average total duration of cases of pleurisy treated by paraceutesis, in connection with other remedies. I will go still farther, and avow my belief that ere long, when we shall puncture *early* after an effusion has occurred, the disease will often be relieved in a much shorter time even than $7\frac{1}{2}$ weeks."

Dr. Bowditch is in favor of medical injections after the operation. He thinks the recent observations and experiments of MM. Boinet and Aran, in Paris, prove conclusively, not merely the safety but the advantage, in some instances, of injections of tr. of iodine.

The paper concludes with a quotation from a letter to Dr. Bowditch "from a gentleman well known in this country and in Europe, and who has had as much experience on this subject as any other individual on either side of the Atlantic." Under the date of June 2d, 1852, this gentleman writes, "It has indeed surprised us as well as yourself, that so simple, so harmless, and so beneficial an operation (when proper precautions are taken, by competent observers,) has been so little regarded in America or England, where it most strangely continues to be esteemed as a most important and serious one." "It may be ineresting to you to know that I have myself been present at, directed, or superintended, at least eighty, and, I quite believe, one hundred operations of paracentesis thoracis" [by puncture with an exploring trocar and the subsequent introduction of a larger one, and without the use of any sucking pump,] "and I never knew it in any of those cases, do any injury; that in a vast majority of these instances, it has been attended with marked benefit; and that in many, where a cure was possible, it has been the important element in effecting that cure."

2. Mr. Tracy relates the case of a child æt. 7, in which paracentesis thoracis was performed for pleuritic effusion with the most beneficial results. He says:

I was first called to see this patient on the 26th of June, 1853, and was told by the mother that he had enjoyed good health until about three months previously, when he caught cold by going into a pond, and since then he had been troubled with dyspnœa, and pain in the left side, and had gradually been declining.

I found him in the following condttion: habit scrofulous, emaciation great, anorexia, pain in the left side, and most marked on full inspiration, dyspnœa, pulse 120, tongue coated. Examination of the chest gave the following signs: dullness over entire left lung, and increased resonance over right; absence of all respiratory sound on the left side, and puerile respiration on the right; point of the heart's impulse full two inches to the right of the mesian line: bulging of left intercostal spaces.

July 2d.—I was called in haste, and found the patient laboring under urgent dyspnœa, and speechless. The pulse was very weak, and according to the nurse, it was imperceptible at the wrist an hour previously. Under the free use of stimulants, and the application to the chest of camphor and ether, he gradually revived.

July 3d.—From this date till the 7th, the patient lay on his back, inclining to the left side, with head raised, and nostrils wildly dilated. The gravity and urgency of the symptoms continued, and he was evidently sinking every hour.

July 7th.—This morning, chloroform having been administered, the operation of puncturing the thorax was performed by Dr. Graves, Dr. Lyford and myself being present. An incision was made with the scalpel between the 6th ahd 7th ribs, and a trocar introduced, through the canula of which a quart of straw-colored serum escaped; and, at each inspiration, air freely entered the wound. On withdrawing the instrument, adhesive plaster with a binder was applied. In the afternoon I saw the patient again, and found his dyspnœa relieved, and his pulse at 110. He said, "I feel much better, and I can breathe easier."

July 8th.—Continues to improve, and amuse himself with his playthings; pulse 120, and tongue less coated; a blister was applied to the side.

July 9th.—Tongue clean and moist, pulse 120, steady.

July 15th.—Pulse 115, tongue clean, appetite good, heart gradually returning to its proper place, and bronchial respiration can be heard in the left lung.

July 22d.—The patient has gradually gained strength and flesh, and now walks out. The left side measures half an inch less in circumference than the right, and the respiration sounds in the left lung are distinctly audible, though faint. He has no cough or dyspnœa, and his appetite is good. There is air yet in the pleural cavity; the position of the heart is nearly natural.

Aug. 1st.—Gains flesh rapidly, pulse 100, the heart in its natural situation, and the function of the lung restored.

